# Synthesis and Characterization of MgO Thin Films Obtained by Spray Technique for Optoelectronic Applications

**DOI:** 10.3390/nano11113076

**Published:** 2021-11-15

**Authors:** Maher Tlili, Chayma Nefzi, Badriyah Alhalaili, Chaker Bouzidi, Lassaad Ajili, Neila Jebbari, Ruxandra Vidu, Najoua Turki Kamoun

**Affiliations:** 1 Laboratoire de Physique de la Matière Condensée, Département de Physique, Faculté des Sciences de Tunis, Université Tunis El Manar, Campus Universitaire, Tunis 2092, Tunisia; maherbargaoui@gmail.com (M.T.); chaimanefzi2021@gmail.com (C.N.); neilajebbari@gmail.com (N.J.); n.najouakamoun@gmail.com (N.T.K.); 2 Nanotechnology and Advanced Materials Program, Kuwait Institute for Scientific Research, P.O. Box 24885, Safat 13109, Kuwait; bhalailil@kisr.edu.kw; 3 Centre National des Recherches en Sciences des Matériaux (CNRSM), Laboratoire de Physico-Chimie des Matériaux Minéraux et Leurs Applications, Borj Cedria Technopark, BP 73, Soliman 8027, Tunisia; chaker.bouzidi1978@gmail.com (C.B.); lassaad.laajili@gmail.com (L.A.); 4 Institut Préparatoire aux Etudes d’Ingénieurs d’El Manar, B.P. 244 El Manar II, Tunis 2092, Tunisia; 5 Faculty of Materials Science and Engineering, University POLITEHNICA of Bucharest, 060042 Bucharest, Romania; 6 Department of Electrical and Computer Engineering, University of California Davis, Davis, CA 95616, USA

**Keywords:** magnesium oxide (MgO) thin film, physical properties, antireflective, photocatalysis application

## Abstract

Magnesium oxide (MgO) thin films with different magnesium concentrations ([Mg^2+^] = 0.05, 0.1, 0.15 and 0.2 mol·L^−1^) in a spray solution have been successfully grown using a spray pyrolysis technique. X-ray diffraction (XRD), Maud software, FTIR spectroscopy, a confocal microscope, Wien2k software, spectrophotometry and a Photoluminescence spectrometer were used to investigate the structural, morphological and optical properties. XRD analysis revealed a better crystalline quality of the MgO thin layer synthesized with [Mg^2+^] = 0.15 mol·L^−1^, which crystallized into a face-centered cubic structure along the preferred orientation (200) lattice plan. The enhancement of the crystalline quality for the MgO thin film ([Mg^2+^] = 0.15 mol·L^−1^) was obtained, which was accompanied by an increment of 94.3 nm of the crystallite size. No secondary phase was detected and the purity phase of the MgO thin film was confirmed using Maud software. From the transmission spectra results, high transparent and antireflective properties of the MgO thin film were observed, with an average transmission value of about 91.48% in the visible range, which can be used as an optical window or buffer layer in solar cell applications. The films also have a high reflectance value in the IR range, which indicates that the highly reflective surface will prevent an increase in surface temperature under solar irradiation, which could be beneficial in solar cell applications. A direct band gap type was estimated using the Tauc relation which is close to the experimental value of 4.0 eV for optimal growth. The MgO material was tested for the degradation of methylene blue (MB), which reached a high photodegradation rate of about 83% after 180 min under sunlight illumination. These experimental trends open a new door for promising the removal of water contaminants for photocatalysis application.

## 1. Introduction

Magnesium oxide (MgO) belongs to the transparent oxide family and crystallizes into a face-centered cubic structure, having a = b = c = 4.217 $\dot{A}$ [1,2]. It is characterized by high transmission values in the visible range near to 90% [3] and a wide direct band gap of 7.8 eV for pure MgO [4]. Heo et al. [5] measured a gap energy lower than 7.8 eV and attributed the low band-gap energies to the existence of defects in type F_S_ and F_B_.

A MgO material has the following main advantages: non-toxicity [6], an abundance of its constituents as well as chemical and physical stability [7]. These properties recommend MgO for a wide range of applications in which its antibacterial [8], antimicrobial [9] and photo-catalytic properties [3,10] could be used. Moreover, MgO can be used for photovoltaic devices [11,12] and gas sensor applications [13]. 

Many experimental techniques have been developed to obtain MgO materials, such as: thermolysis of an ultrathin Mg(OH)_2_ precursor under a dynamic vacuum to obtain ultrathin single-crystalline MgO nanosheets with a preferential orientation stacked by (111) planes [6], sol-gel [14,15,16], chemical vapor deposition (CVD) [17], pulsed laser deposition [18], reactive sputtering [19], laser ablation [20], metal organic molecular beam epitaxy [21] and a chemical spray pyrolysis technique [3,22]. Currently, heavy environmental pollution has motivated researchers to seek new treatments, such as the extractive–pyrolytic method [23], to remove pollutants and protect the environment. For these reasons, semiconductor materials are involved in the photocatalysis process for the degradation of organic pollutants, which are hazardous to human health and harmful for the environment. In this work, many experimental techniques and tools are used to investigate the magnesium oxide thin film, such as X-ray diffraction (XRD), FTIR spectroscopy, Maud software, confocal microscopy, spectrophotometry and photoluminescence spectrometry. The main goal of this study is to investigate the physical behaviors of sprayed magnesium oxide thin films with different magnesium concentrations in the sprayed solution ([Mg^2+^] = 0.05, 0.1, 0.15 and 0.2 mol·L^−1^) and look for possible optoelectronic applications. 

## 2. Materials and Methods

MgO thin layers were synthesized on glass substrates using the liquid-phase spray pyrolysis technique. Before the deposition process, all glass substrates were cleaned in an ultrasonic bath containing double-distilled water. Magnesium II chloride hexahydrate (MgCl_2_, 6H_2_O, 99%) was purchased from AppliChem (Council Bluffs, IA, USA). MgCl_2_ was dissolved in 100 mL of bi-distilled water. This solution, which contained the precursor, was sprayed onto preheated substrates as fine droplets by means of compressed air as a carrier gas. During the deposition process, the substrate temperature was maintained at 450 °C and the solution flow rate was kept at 10 mL/min. In this work, we varied magnesium concentrations ([Mg^2+^] = 0.05, 0.1, 0.15, 0.2 mol·L^−1^) to study the physical properties of the MgO material. 

The crystalline structure of the thin films was studied by XRD using an X-ray diffractometer with a 1.5418 Å Cu-Kα ray (automated Bruker D8 apparatus, Karlsruhe, Germany). The experimental XRD spectra were compared with the Maud software. FTIR spectroscopy (type VERTEX80 spectrometer for 400–4000 cm^−1^, Billerica, MA, USA) was performed to identify the existence of different molecules and ions on the sample surface, and the existence of MgO in particular. Morphological analysis was performed using a confocal microscope called “SENSOFAR”. Optical measurements were performed using a Perkin Elmer Lambda 950 spectrometer (Bridgeport, CT, USA). The type and value of the band gap were calculated using the Tauc relation. The photocatalysis process of the MgO thin layer was tested by degrading aqueous methylene blue (MB, 95%, from Sigma Aldrich, Bengaluru, Karnataka, India) under sunlight irradiation at room temperature and ambient air. Thus, to prepare the aqueous MB solution, 5 mg of MB powder was dissolved in double-distilled water (from Bi-distiller water GFL, Burgwedel, Germany) to reach 10^−5^ M of dye solution. Next, each sample was immersed in 20 mL of aqueous MB solutions. Then, all solutions were placed in the dark for 30 min before exposing them to light in order to achieve the adsorption–desorption equilibrium state. The degradation of MB dye was measured at different periods of time (from 30 min to 3 h, in steps of 30 min), using a Perkin Elmer Lambda 950 spectrophotometer.

## 3. Results and Discussion

### 3.1. XRD Analysis

**Figure 1:**
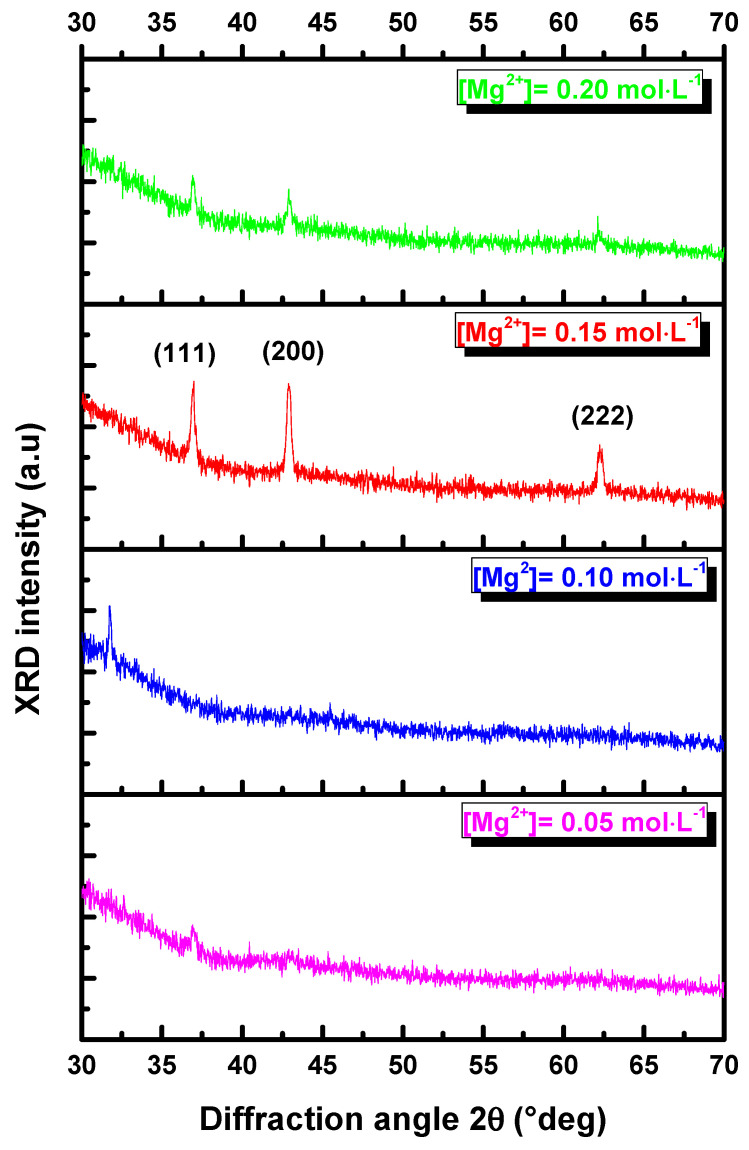
X-ray spectra of MgO thin films deposited on glass substrates for different concentrations ([Mg^2+^] = 0.05; 0.10; 0.15 and 0.20 mol·L^−1^).

In order to study the effect of magnesium concentrations on the structural property of MgO thin films, different amounts of Mg were used ([Mg^2^^+^] = 0.05, 0.1, 0.15 and 0.2 mol·L^−1^). The diffractograms obtained by scanning the 2θ range between 20° and 80° are presented in Figure 1. The XRD patterns of the synthesized MgO obtained for [Mg^2^^+^] = 0.05–0.1 mol·L^−1^ show low-intensity peaks. The XRD scans of the MgO thin films present characteristic peaks that correspond to (111), (200) and (222) plans, which are attributed to the face-centered cubic structure (JCBD card No# 650476) with space group fm$\bar{3}$m. The onset of MgO polycrystalline material was observed at [Mg^2^^+^] = 0.15–0.2 mol·L^−1^. It is observed that the crystallinity increases with the increase in the intensity of the (200) preferred orientation for an Mg concentration of 0.15 mol·L^−1^. However, an increase in Mg concentration to 0.2 mol·L^−1^ results in a slight deterioration of crystallinity. The crystallite size D (nm) was calculated using the Debye–Scherrer equation [3,24]:

(1) $$D=\frac{k\lambda }{\beta \cos\left(\theta \right)}$$

where the constant *k* equals 0.9, the wavelength of the incident X-ray is λ=1.54 nm, β is the full width at half-maximum of the diffraction peak and 2θ is the position of the preferred orientation (200).

Table 1 summarizes the calculated crystallite size (D), dislocation density (δ_dis_ = $\frac{1}{D^{2}}$) per unit area and the strain ($\varepsilon =\frac{\beta \cos\theta }{4}$) of MgO thin films synthesized with different Mg concentrations. The results of these calculations show that D increases with increasing magnesium concentrations, reaching the highest value of about 9 nm at [Mg^2+^] = 0.15 mol·L^−1^. The maximum size of MgO crystallite (D = 9 nm) obtained by the spray technique is less than D = 50 nm, obtained by Demirci et al. [10], who grew MgO by flame spray pyrolysis, but larger than D = 7.8 nm, obtained by Poonguzhali et al. [2] for MgO nanorods. It is worth mentioning that this trend is correlated with the improvement in crystalline quality, as seen in Figure 1. Moreover, Table 1 shows that the dislocation density $\delta_{\mathrm{dis}}$ was reduced from 35.45 × 10^15^ cm^−2^ for [Mg^2+^] = 0.1 mol·L^−1^ to 12.1 × 10^15^ cm^−2^ for [Mg^2+^] = 0.15 mol·L^−1^. A similar trend was observed for the strain, which varied in the range of 0.065 × 10^−3^–0.043 × 10^−3^%.

### 3.2. Rietveld Analysis

**Figure 2:**
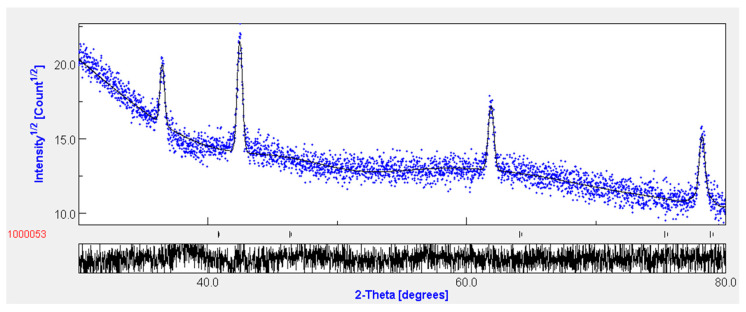
Rietveld refinement of optimum MgO film grows by spray pyrolysis with [Mg^2+^] = 0.15 mol·L^−1^.

In order to refine the experimental XRD spectra and confirm the face-centered cubic structure of the MgO material, we used the Maud software (Materials Analysis Using Diffraction) based on the Rietveld analysis [3]. Additionally, we could estimate the lattice parameter and crystallite size and we could extract the secondary phases, which may exist along with the MgO phase. Figure 2 illustrates the Rietveld refinement of the XRD spectrum for the MgO thin layer prepared using a spray solution containing [Mg^2^^+^] = 0.15 mol·L^−1^. The results show that no secondary phase exists along with the MgO phase, which proves the purity of the MgO material. We obtained a high goodness-of-fit (GOF) equal to 1.05. The crystallite size value was about 11.02 nm, which is very close to the experimental value obtained in Table 1. After applying the fitting analysis, we obtained a lattice parameter equal to a = 4.52 $\dot{A}$, which is in good agreement with the JCPDF card No. #650476. 

### 3.3. FTIR Spectra 

**Figure 3:**
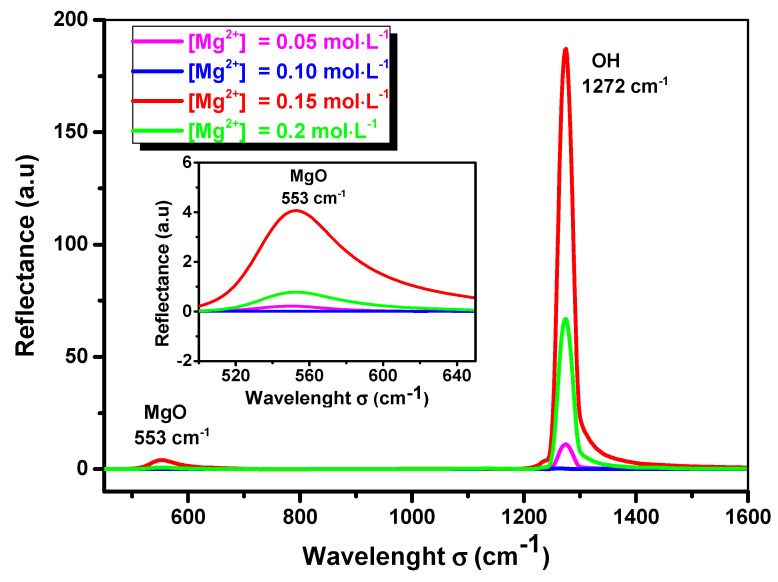
FTIR spectra of sprayed MgO thin films deposited with [Mg^2^^+^] = 0.05; 0.10; 0.15 and 0.20 mol·L^−1^.

The FTIR spectra of the MgO thin films deposited with [Mg^2+^] = 0.05; 0.10; 0.15 and 0.20 mol·L^−1^ show two peaks located at 553 cm^−1^ and 1272 cm^−1^ (Figure 3). The peak at 553 cm^−1^ indicates the stretching vibration of MgO which was reported by Ashok et al. [24], who obtained MgO nanoparticles using a microwave irradiation technique and found a vibrational of MgO at 588 cm^−1^; they also mentioned that MgO has a stretching vibration in the range of 550–670 cm^−1^. Tlili et al. [3] prepared MgO thin films by a spray pyrolysis technique and they found a vibrational of MgO at 459 cm^−1^. Kandiban et al. [25] synthesized MgO nanoparticles using co-precipitation and a hydrothermal method and found a vibrational of MgO at 548 cm^−1^.

The peak located at 1272 cm^−1^ was assigned to the hydroxyl group OH, which was reported by Moses et al. [22], who found the peak at 1228 cm^−1^, and by Devaraja et al. [26], who mentioned that the hydroxyl group of water has an absorption in the range of 1300–1800 cm^−1^. The spectrum presented in Figure 3 shows no other elements or impurities on the surface, such as carbon monoxide CO [24,25] or H^−^, CO_3_^2−^ (located at 1076, 1435 cm^−1^) [27].

As the size of the particle decreases, the resolution of the vibrational bands is better resolved [28]. The peak intensity of MgO with [Mg^2+^] = 0.15 mol·L^−1^ is the largest of all peaks in the FTIR spectrum, which agrees well with the largest crystallite size obtained by XRD, and is presented in Table 1.

### 3.4. Surface Morphology 

**Figure 4:**
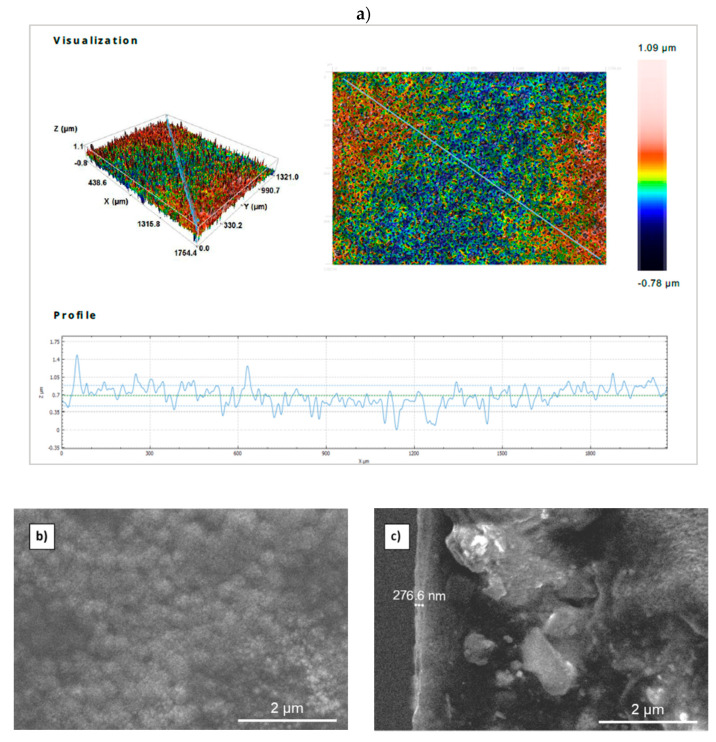
Confocal microscopy (**a**) and SEM images (top (**b**) and cross-section (**c**) views) of the MgO thin layer synthesized using [Mg^2+^] = 0.15 mol·L^−1^.

To study the morphological aspects of the MgO thin layer, a 3D confocal microscope (1764 × 1321 μm) was used, as shown in Figure 4a. The micrograph clearly shows the uniform and dense aggregation of particles that have practically the same size. Moreover, the diagonal profile line of the scanned surface area gives an average thickness of about 0.4 μm, which is close to the value calculated from the double weight that equals 0.36 μm for optimum growth. It is worth mentioning that the MgO thin film obtained using [Mg^2^^+^] = 0.15 mol·L^−1^ exhibits a rough surface morphology. This observation is very important, because a rough surface means a large contact surface, which is a beneficial feature in photocatalysis, humidity and sensor applications. The surface parameters of the MgO material obtained from the confocal microscope data show that the arithmetical mean height (Sa) value equals 0.15 µm and the root mean square (Sq) equals 0.19 μm. It is clear that the MgO thin film is grown using [Mg^2+^] = 0.15 mol·L^−1^ with high roughness, which makes it a promising candidate for gas sensor applications as well as for photocatalysis water treatment [3].

Figure 4b,c show the SEM images (top and cross-section views of the MgO thin film obtained using [Mg^2+^] = 0.15 mol·L^−1^. These images show a surface morphology characterized by spherical, droplet-like particles and a smooth, continuous film without any cracks. The SEM image of the cross-section view (Figure 4c) shows that the film has a thickness of 276.6 nm. We also performed a double weighting method to assess the thickness of the layers. Using a precision balance of 0.0001 g, the film thickness was estimated to be 290 ± 25 nm, which agrees well with the film thickness measured by SEM. 

### 3.5. Optical Analysis 

**Figure 5:**
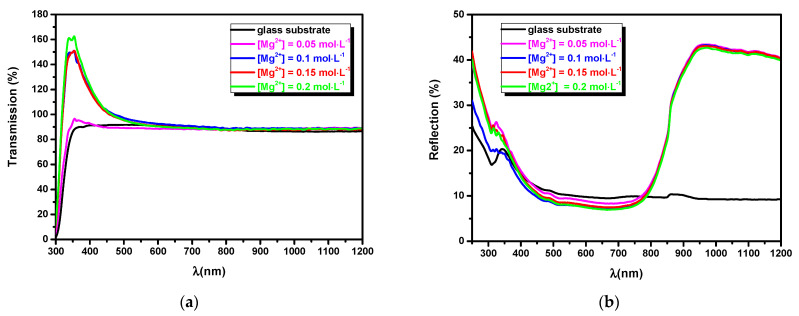
(**a**) Transmission and (**b**) reflectance spectra of sprayed MgO thin films grown for different concentrations [Mg^2+^] = 0.05; 0.10; 0.15; and 0.20 mol·L^−1^.

Using various magnesium concentrations in the spray solution, both morphological and structural behaviors will induce significant changes in the optical properties of MgO. Figure 5 displays the transmission T (%) and the reflectance R (%) spectra of the MgO thin films in UV-Vis-IR regions. High transmission values in the visible range for all samples recommend the MgO thin film as an optical window or buffer layer in solar cell devices. Figure 5a shows that T (%) values increase as the Mg concentration increases. Generally, the enhancement of T (%) could originate from reduced scattering effects, the enhancement of crystallinity and structural homogeneity, as reported by [29,30]. On the other hand, a particular behavior was observed, i.e., T (%) exceeded 100% in the UV range around λ = 350 nm. These results could be explained by the fact that the MgO thin film adsorbed H_2_O from the atmosphere. Thus, the water droplets which exist in the ambient air were well retained on the rough surface of the film, which in turn increased the transmission values, as mentioned earlier [3]. This observation indicates that the MgO thin film could be used as a humidity sensor.

The reflectance spectra presented in Figure 5b show low intensity values close to 8% in the visible range for the MgO film obtained from a spraying solution containing [Mg^2^^+^] = 0.15 mol·L^−1^. In addition, high reflection values of 45% were obtained in the near-infrared region. These results indicate that a highly reflective surface will prevent an increase in surface temperature under solar irradiation, which could prove beneficial in solar cell applications. On the other hand, point defects may affect the optical absorption, as was observed in the MgO single crystal [2].

From the transmission T (λ) and reflectance R (λ) values, the absorption coefficient (α) can be analyzed using the following formula [30]:

(2) $$\alpha =\frac{1}{e}\;\mathrm{Ln}\left(\frac{{\left(1-R\right)}^{2}}{T}\right)$$

where e is the film thickness determined by the double weighting method. 

**Figure 6:**
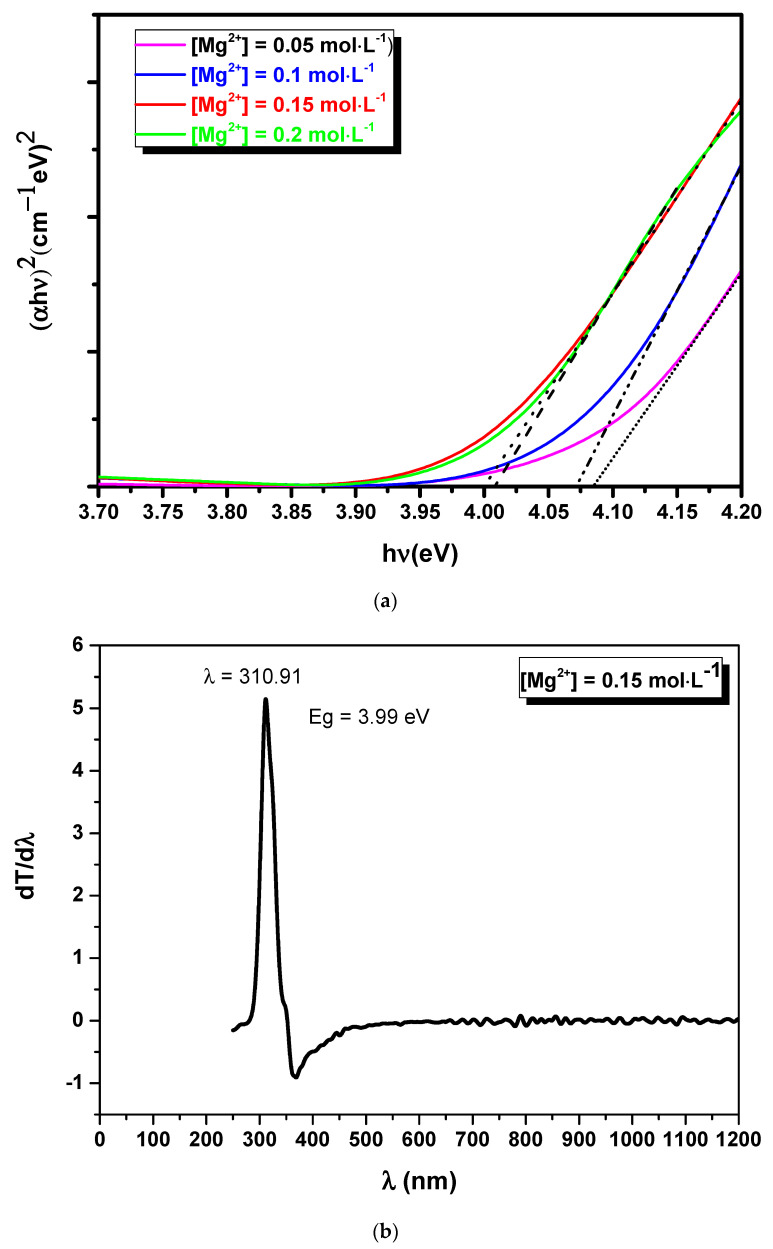
Band gap energy of MgO thin layers deposited by spray for [Mg^2+^] = 0.05; 0.10; 0.15; and 0.20 mol·L^−1^ (**a**) and the value of the band gap energy of MgO for the [Mg^2+^] = 0.15 mol·L^−1^ concentration obtained from the graph of the derivative of T with respect to λ as a function of λ (**b**).

The value and the type of the band gap energy were obtained from the Tauc relation,

(3) $$(\alpha h\nu )=B{\left(h\nu -\mathrm{Eg}\right)}^{n}$$

where ν = c/λ, h = 6.62 × 10^−34^ J.s and B is a constant that depends on the transition probability. Experimental energy band gaps of MgO thin films were estimated by plotting (αhν)^2^ versus hν (eV), as shown in Figure 6. The intersection of the quasi-linear part of curve with the x-axis shows that the MgO thin film obtained using [Mg^2^^+^] = 0.15 mol·L^−1^ has a large energy band gap of 4 eV. Table 2 summarizes the energy band gap values. These values are lower than the band gap energy of pure and bulk MgO (7.8 eV). The large difference in band gap energy is attributed to the presence of default sites.

**Figure 7:**
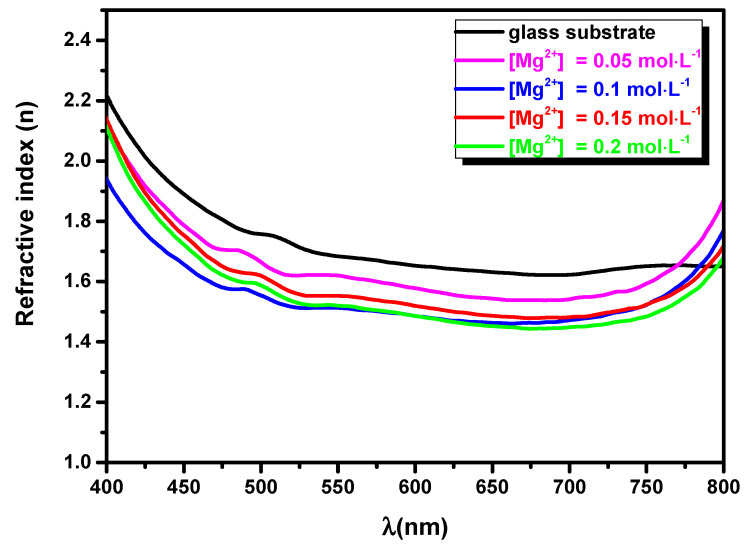
Variation in the refractive index (n) for MgO layers synthesized by spray for [Mg^2+^] = 0.05; 0.10; 0.15; and 0.20 mol·L^−1^.

The refractive index (*n*) was calculated using the following relation [31]:

(4) $$n=\frac{1+[1-{\left(\frac{1-R}{1+R}\right)}^{2}\;{\left(1+\left(\frac{\lambda .\alpha }{4\pi }{)}^{2}\right)\right]}^{\frac{1}{2}}}{\frac{1-R}{1+R}}$$

where: α and *R* are the absorption coefficient and the reflection, respectively. Figure 7 illustrates the refractive index versus the wavelength (*λ*) for MgO thin layers obtained from spray solutions with different magnesium concentrations. Figure 7 shows that n varies in the range from 1.5 to 2.2. According to Table 1 and Table 3, the variation in the refractive index follows the variation in the film thickness and the reduction in band gap energy, especially for [Mg^2+^] = 0.15 mol·L^−1^. 

### 3.6. Photoluminescence (PL) 

We performed photoluminescence measurements at 200 and 220 nm on the MgO thin layer obtained using a concentration of 0.15 mol·L^−1^ Mg in the spray solution. When the film was excited with a wavelength of 220 nm (Supplementary Material, Figure S1), we observed the presence of emission peaks at 3.35, 3.16 and 2.32 eV, which were attributed to defect center F2, F + and F, respectively, according to Kotomin et al. [32].

**Figure 8:**
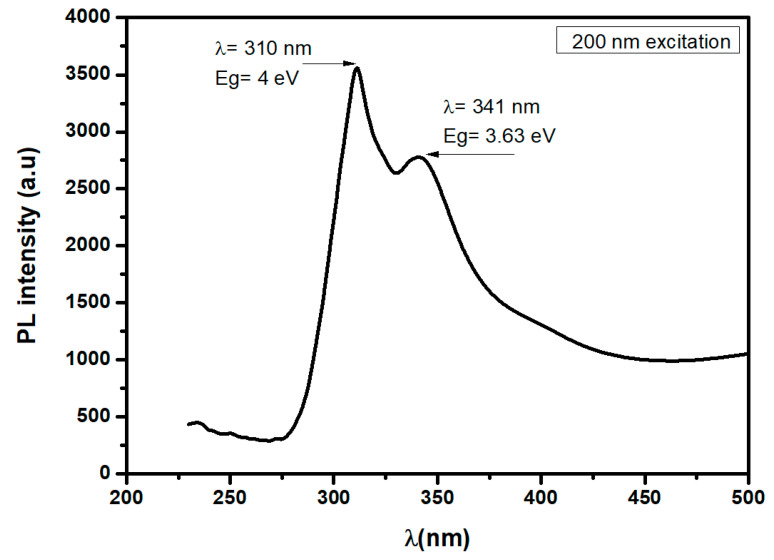
PL emission spectrum for 200 nm excitation of MgO thin films prepared with [Mg^2+^] = 0.15 mol·L^−1^.

Figure 8 displays the photoluminescence spectra of MgO thin films synthesized with [Mg^2+^] = 0.15 mol·L^−1^ excited with a wavelength of 200 nm, where only two emission peaks were observed at 310 nm and 341 nm, which correspond to 4 and 3.63 eV, respectively. These results agree with the value of the band gap energy of MgO for the [Mg^2+^] = 0.15 mol·L^−1^ concentration obtained from the graph of the derivative of T with respect to λ as a function of λ in Figure 6.

### 3.7. Photocatalysis Process 

**Figure 9:**
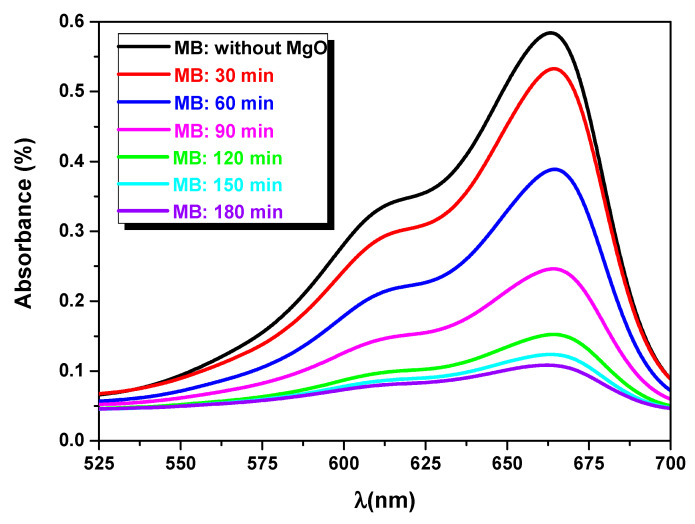
The temporal change in the original MB solution (0 min) and with MgO thin films under sunlight irradiation for different times (30, 60, 90, 120, 150 and 180 min).

Figure 9 shows the photocatalytic activity of MgO ([Mg^2+^] = 0.15 mol·L^−1^) thin film at different times (i.e., 30, 60, 90, 120, 150 and 180 min) under sunlight illumination. The MgO thin layer effectively decomposed the aqueous methylene blue (MB) after 180 min. In order to study the photocatalytic degradation, we calculated the photodegradation rate of MgO using the following expression [3]:

(5) $$\mathrm{Photodegradation}\;\mathrm{rate}=\frac{C_{0}-C}{C_{0}}\times 100$$

where *C* and *C*_0_ are the absorbance values of the MB dye solution with and without the MgO sample, respectively. 

**Figure 10:**
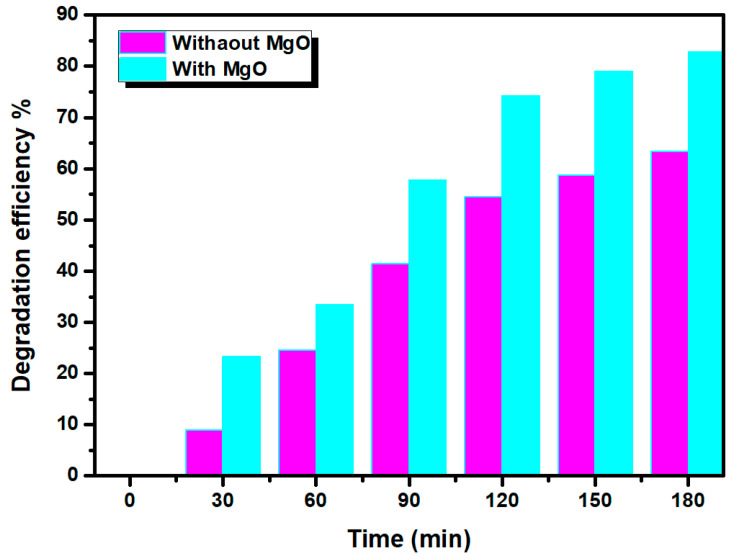
Photodegradation rate variation in MB for two MB solutions: the one contained the optimum MgO (blue) and the other of original solution (pink) for different times.

The photodegradation rate of MB dye under sunlight irradiation at different times is illustrated in Figure 10. The value calculated for the photodegradation rate was about 83% after 180 min. This result could be related to the higher surface roughness of the MgO thin layer obtained with [Mg^2+^] = 0.15 mol·L^−1^. 

The kinetic constant was investigated from the following expression [33]:

(6) $$\frac{\mathrm{dC}}{\mathrm{dt}}=-kC$$

**Figure 11:**
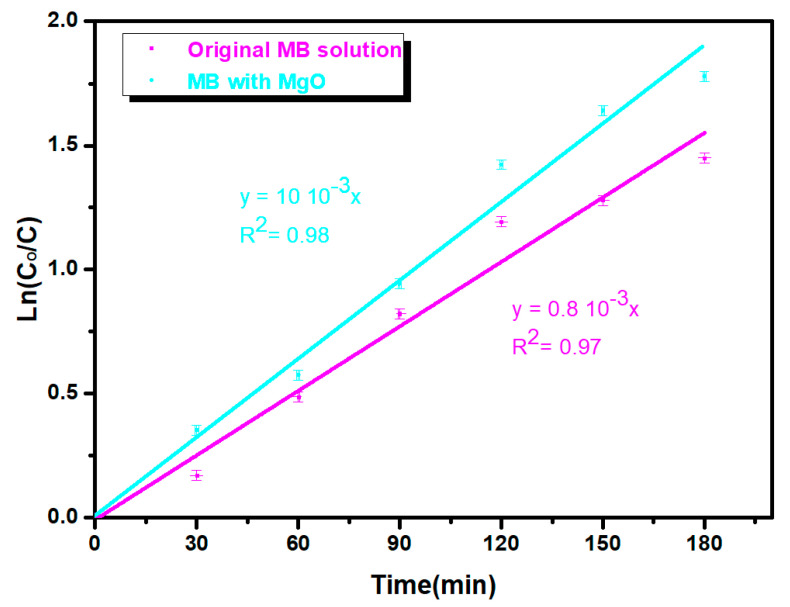
Variation in Ln (C_0_/C) of original MB solution (pink) and with MgO (blue) thin films under sunlight irradiation for different times (30, 60, 90, 120, 150 and 180 min). Error bar is 0.02.

Figure 11 displays the kinetic constant of the MgO thin film, which confirms the assumption of the first order of the kinetic constant. Plotting the Ln ($\frac{C_{0}}{C}$) curve as a function of time, the k_1_ value of the MB solution without sample (Ln($\frac{C_{0}}{C}$) = k_1_.t) and k_2_ with the MgO sample (Ln($\frac{C_{0}}{C}$) = k_2_.t) were equal to 0.17 10^−3^ s^−1^ and 0.13 10^−3^ s^−1^, respectively. The kinetic constant (k = 0.17 10^−3^ s^−1^ = 0.01 min^−1^) is much smaller than k = 0.30 min^−1^, obtained by Demirci et al. [10], who used UV light. In our experiments, we used the natural solar light to break down the MB, which has an intensity lower than that of a UV lamp. We can improve the kinetic constant by doping the MgO. In fact, doping or co-doping MgO with transition metals or with rare earth elements also increases the surface roughness, which increases the specific contact surface between the MB solution and the MgO material, increasing the photocatalytic efficiency, as confirmed by Kamoun et al. [34] for the MoO_3_ films co-doped with Fe or Co.

We note that k_2_ is greater than k_1_, confirming the efficiency of the MgO material to decompose the MB dye. This study suggests that the MgO thin film is a good photocatalyst for removing organic pollutants in water.

## 4. Conclusions 

In summary, MgO thin layers were synthesized by a spray pyrolysis technique at different concentrations, i.e., 0.05 ≤ [Mg^2+^] ≤ 0.20 mol·L^−1^. It was found that the best results were obtained for [Mg^2+^] = 0.15 mol·L^−1^. The XRD and Maud software revealed that the good MgO phase crystallized into a face-centered cubic structure along (200) with preferential orientation. The energy band gap value of MgO was estimated by the Tauc relationship, giving an energy gap value near to 4 eV for MgO obtained using [Mg^2+^] = 0.15 mol·L^−1^. Noticeable optical transmission behavior of the MgO thin films includes that the T(%) values exceeded 85% in the visible region and were higher than 100% in the UV region. Owing to its intrinsic properties, we observed that MgO obtained by an inexpensive method such as spray pyrolysis has a high absorption of water molecules, which was also confirmed by FTIR analysis. Because of these characteristics, the MgO thin film can be used as a humidity sensor. Additionally, the MgO thin film presents good efficiency for the degradation of MB dye under solar irradiation, which recommends this material for photocatalysis water treatment.

## Figures and Tables

**Table 1:** Structural parameters of MgO thin layers grown by spray for different magnesium concentrations: [Mg^2+^] = 0.05; 0.10; 0.15; 0.20 mol·L^−1^.

[Mg^2+^] (mol·L^−1^)	2θ (°)	Crystallite Size D (nm)	Dislocation Density δ_dis_ (10^15^ cm^−2^)	Micro Strain *ε* 10^−3^ (%)
0.05	-	-	-	-
0.10	42.38	5.31	35.5	0.065
0.15	42.67	9.10	12.1	0.038
0.20	42.50	8.00	15.6	0.043

**Table 2:** Band gap energies of MgO elaborated for different concentrations [Mg^2+^] = 0.05; 0.10; 0.15; and 0.20 mol·L^−1^.

[Mg^2+^] (mol·L^−1^)	0.05	0.10	0.15	0.20
band gap energy (eV)	4.08	4.07	4.00	4.01

**Table 3:** Film thickness of MgO synthesized with [Mg^2+^] = 0.05; 0.10; 0.15; and 0.20 mol·L^−1^.

[Mg^2+^] (mol·L^−1^)	0.05	0.1	0.15	0.2
Thickness (µm)	0.11	0.22	0.29	0.40

## Data Availability

The data presented in this study are available on request from the corresponding author. The data are not publicly available due to funder retention policies.

## Supplementary Materials

The following are available online at https://www.mdpi.com/article/10.3390/nano11113076/s1, Figure S1. PL emission spectrum for 220 nm excitation of MgO thin films prepared with [Mg^2+^] = 0.15 mol·L^−1^.
