# Supplementary material for: Synthesis and Characterization of MgO Thin Films Obtained by Spray Technique for Optoelectronic Applications
Source: Nanomaterials (Basel). 2021 Nov 15;11(11):3076. doi: 10.3390/nano11113076 (PMC8618750; doi:10.3390/nano11113076)
Supplement: Supplementary file 1 [file nanomaterials-11-03076-s001.zip › nanomaterials-1379990-supplementary.pdf]

nanomaterials

Supplementary Materials: Synthesis and Characterization of MgO Thin Films Obtained by Spray Technique for Optoelectronic Applications

Maher Tlili [1], Chayma Nefzi [1], Badriyah Alhalaili [2], Chaker Bouzidi [3], Lassaad Ajili [3], Neila Jebari [4], Ruxandra Vidu [5,6,*] and Najoua Turki Kamoun [1]

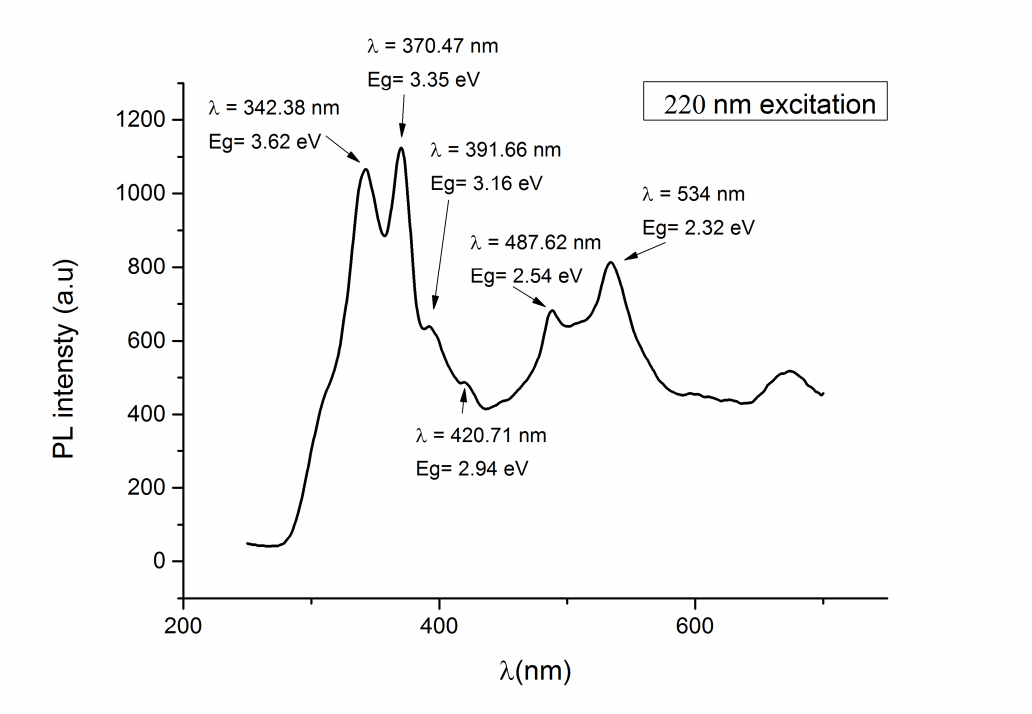

Figure S1. PL emission spectrum for 220 nm excitation of MgO thin films prepared with $[Mg^{2+}] = 0.15$ mol·L^{-1}.

The emitted radiations are presented in figure 8. We observe the presence of peaks of emissions at: 3.35 eV, 3.16 and 2.32 which are attributed to defect center of type F2, F^+ and F, respectively according to Kotomin et al [29].
